# Climate change and budget cuts: Facing the *Aedes* and *Anopheles* challenges together

**DOI:** 10.5281/zenodo.21456545

**Published:** 2026-07-20

**Authors:** Michael MacDonald

**Affiliations:** 1 Public Health Entomologist, Catonsville, Maryland. USA.

## Abstract

Funding and organisational barriers between *Aedes* and *Anopheles* hamstring vector control adaptation to this new era of climate change and restricted public funding. *Aedes*-borne diseases never got the attention or funding like malaria and are now exploding across Asia and the Americas with devastating economic impact. Malaria elimination progress has stalled, and in several contexts reversed, due to sudden donor funding restrictions. There are both tactical and strategic ways the two vector control efforts can work together to meet these challenges. Tactically, these include insecticide resistance management, residual spraying, technology for larval source management, spatial emanators and surveillance. Strategically, community engagement, urbanisation and climate change, and financing/private sector engagement. Malaria and *Aedes*-borne diseases have separate support networks and ‘communities of practice’. Now is the time to recognise mutual tactical and strategic benefits, break down the artificial barriers between these two worlds, and face our common challenges together.

In this time of crisis, it’s time to break down the donor-imposed barriers between malaria and *Aedes*-borne diseases and recognise both the tactical and strategic mutual benefits of joining forces.

Described in 1999 as ‘An Environmental Plague for the New Millennium’ [1] dengue transmission, fuelled by climate change, urbanisation and population mobility, has exploded with now half the world's population at risk and 14 million cases reported in 2024. Along with chikungunya, Zika and yellow fever, dengue is readily transmitted by the urban, domestic *Aedes aegypti* and highly invasive *Ae. albopictus*, who has proliferated in the USA since first introduced in the 1980’s and is now rapidly expanding through Europe and China, where it caused a crippling chikungunya outbreak in 2025.

*Aedes*-borne diseases, with the brief exception of Zika, don’t get the attention or the funding of the big childhood killers, malaria, respiratory and diar-rhoeal diseases, and certainly not HIV. Donors did not appreciate the Return on Investment (RoI) like malaria where an investment in nets, diagnostics and drugs will return an expected reduction in illness and death. Dengue and chikungunya are not so simple. Bednets have very limited impact (*Aedes* feeds during the day) and diagnostics, therapeutics and vaccines are much more complex. Two dengue vaccines completed clinical trials and received international approval. A third vaccine rollout in Brazil was recently suspended [2]. Moreover, the economic impact of dengue and chikungunya may be far greater and insidious than the headline-grabbing child mortality figures for these other disease-prevention investments.

Globally, *Aedes*-borne diseases cost US$3.3 billion annually; total costs can surge significantly higher during peak outbreak years, causing tens of billions in financial strain. The 2016 Zika outbreak in Latin America and the Caribbean cost $3.5b [3]. At the family level, a cost-of-illness study in Bangladesh showed the overall out of pocket expenditure for dengue treatment was 56% of monthly household income. However, in the poorest quintile, it exceeded 139% of total household income, plunging families into debt. It’s going to get worse.

Global warming is estimated to have caused 18% of historical dengue. With future warming this could further increase incidence by 49% to 76% by midcentury, with cooler regions projected to double in incidence; for Bolivia, population-weighted average dengue is projected to in-crease by 811% [4].

For malaria, financial stress, drug and insecticide resistance, and conflict and climate-induced humanitarian emergencies have stalled and, in many cases, reversed the path to malaria elimination. When USAID was being dismantled in March 2025, Nicholas Enrich, Acting Assistant Administrator for Global Health issued a memo estimating the budget cuts would result in an additional 12.5-17.9 million malaria cases and an additional 71,000-166,000deaths (39.1% increase) annually; by the weekend he was put on administrative leave [5].

## Tactical mutual benefits

### Insecticide Resistance monitoring and management

*Aedes* lags far behind *Anopheles* as tracked in the WHO Threats Map. The 2013 WHO Global Plan for Insecticide Resistance Management, GPIRM, long overdue for a makeover, may soon be revised and expanded. GPIRM-II is expected to include Aedes, supporting advocacy and best practices for national implementation, including surveillance and multi-sector resistance management among *Aedes*.

### Indoor Residual Spraying (IRS)

Implementation of IRS for malaria is rapidly declining. While implementation strategy revisions may make IRS more cost-competitive with other malaria interventions, there is great room for expansion in ‘new routes to market’ and targeted residual spraying for *Aedes* [6].

### Larval Source Management (LSM)

The expanded use of new technologies, including Artificial Intelligence for targeting, implementation and monitoring LSM for *Anopheles* already overlaps with *Aedes* in the response to invasive *An. stephensi*. There is much more to learn from each other to innovate and optimize.

### Novel approaches and progress

There are several new tools and approaches developed for *Anopheles* being expanded for *Aedes*. Foremost are spatial emanators. While much of the current focus is on public sector programme delivery for malaria, their proven impact on dengue and Zika, opens a huge opportunity for do-it-yourself rapid deployment as well as access through the commercial sector [7].

### Surveillance

While the specific collection methods may differ, there can be tremendous cross-learning between entomological surveillance of *Anopheles* and *Aedes*. Several countries have made significant advances in *Aedes* surveillance, information management, targeting, use of Artificial Intelligence and outbreak prediction that could be shared [8].

## Strategic mutual benefits

### Community engagement

As a bottom-up, household and community based response, minimally funded dengue pro-grammes have developed legislative and community-engagement processes far better than malaria. These include legislation and public works to reduce larval habitats, citizen science, social media, and youth engagement initiatives the malaria world should learn from [9].

### Linking to the Urban Health and Climate Change agendas

Urbanisation and climate change are having a significant impact on *Aedes*-borne diseases [10]. As the malaria world responds to invasive *An. stephensi* and the more general increasing threats of malaria in the built environment, there is a lot to learn from the *Aedes* response. The Roll Back Malaria Healthy Cities Healthy People initiative aims to put city and local government leaders at the heart of national and global health debates, often with dengue and chikungunya as the entry point [11].

### Financing, including private sector

On the other side, malaria has demonstrated ‘the business case’ for private sector investment for malaria control in their communities [12]. These can be a blueprint for RoI for *Aedes* control, especially for the tourism sector who have seen catastrophic financial losses from chikun-gunya and Zika outbreaks [13,14].

## Roll Back Mosquitoes: expanding and linking platforms

Malaria has the *RBM Partnership*, including the *Vector Control Working Group* [13]; the *African Leaders Malaria Alliance* [14]; the *Asia Pacific Leaders Malaria Alliance* [15], and the *Asia Pacific Malaria Elimination Network* [16], again with a *Vector Control Working Group* [17]. There are opportunities to formally expand and collaborate with the parallel networks for *Aedes*-borne diseases. These include *Asia Dengue Voice & Action* [18], *International Society for Neglected Tropical Diseases* [19], the *Collective Action on Dengue* [20], and the more recently formed *Global Dengue and Aedes-transmitted Diseases Consortium* [21].

WHO itself has combined the Global Malaria Programme and Neglected Tropical Diseases to form the Department of Malaria and Neglected Tropical Diseases (MNT), issuing integrated guidance documents. Now is the time for international donors and the networks they support to do the same, recognise the mutual tactical and strategic benefits, break down the artificial barrier between these two worlds and face our common challenges together.
